# Anabolic Therapies in Osteoporosis and Bone Regeneration

**DOI:** 10.3390/ijms20010083

**Published:** 2018-12-26

**Authors:** Gabriele Russow, Denise Jahn, Jessika Appelt, Sven Märdian, Serafeim Tsitsilonis, Johannes Keller

**Affiliations:** 1 Center for Musculoskeletal Surgery, Charité—Universitätsmedizin Berlin, corporate member of Freie Universität Berlin, Humboldt-Universität zu Berlin, and Berlin Institute of Health, 13353 Berlin, Germany; gabriele.russow@charite.de (G.R.); denise.jahn@charite.de (D.J.); jessika.appelt@charite.de (J.A.); sven.maerdian@charite.de (S.M.); serafeim.tsitsilonis@charite.de (S.T.); 2 Julius Wolff Institute for Biomechanics and Musculoskeletal Regeneration, Charité—Universitätsmedizin Berlin, corporate member of Freie Universität Berlin, Humboldt-Universität zu Berlin, and Berlin Institute of Health, 13353 Berlin, Germany; 3 Berlin Institute of Health, 13353 Berlin, Germany

**Keywords:** osteoporosis, anabolic therapy, bone regeneration, parathyroid hormone, sclerostin, romosozumab, denosumab

## Abstract

Osteoporosis represents the most common bone disease worldwide and results in a significantly increased fracture risk. Extrinsic and intrinsic factors implicated in the development of osteoporosis are also associated with delayed fracture healing and impaired bone regeneration. Based on a steadily increasing life expectancy in modern societies, the global implications of osteoporosis and impaired bone healing are substantial. Research in the last decades has revealed several molecular pathways that stimulate bone formation and could be targeted to treat both osteoporosis and impaired fracture healing. The identification and development of therapeutic approaches modulating bone formation, rather than bone resorption, fulfils an essential clinical need, as treatment options for reversing bone loss and promoting bone regeneration are limited. This review focuses on currently available and future approaches that may have the potential to achieve these aims.

## 1. Introduction

Osteoporosis represents a polygenetic, environmentally modifiable bone disease, which often results in fragility fractures and poses a high risk of fractures in low impact trauma. Furthermore, the molecular perturbations leading to osteoporosis are also associated with delayed fracture healing and impaired bone regeneration. Based on a steadily increasing life expectancy in modern societies, the worldwide implications of osteoporosis and impaired bone healing are tremendous. Therefore, the clinical need to reverse bone loss, to stimulate bone formation and to boost bone regeneration is increasing and has become a crucial challenge for professional health care providers. A range of drugs approved by the United States Federal Drug Administration (FDA), which work by inhibiting bone resorption, are available for the prevention and treatment of osteoporosis. These substances including bisphosphonates, the monoclonal antibody denosumab and selective oestrogen receptor modulators, only inhibit the breakdown of bone but do not stimulate the formation of new bone. Research in the last decades has revealed several pathways that stimulate bone formation and could be applied to treat both osteoporosis and impaired fracture healing. This review focuses on currently available and future approaches that may be employed to target bone formation and bone regeneration in every day clinical practice.

## 2. Bone Turnover—Osteoporosis

Skeletal tissue represents a highly dynamic tissue that continues to change throughout a lifespan. This process of skeletal turnover is called bone remodelling and is required to protect the structural integrity of bone tissue and to contribute metabolically to the body’s balance of calcium and phosphate. Remodelling includes the resorption of old or damaged bone (bone resorption), which is followed by the formation of new bone (bone formation). In bone tissue, three different and highly specialized cell types are thought to be responsible for the resorption and formation phases of bone remodelling.

**Figure 1:**
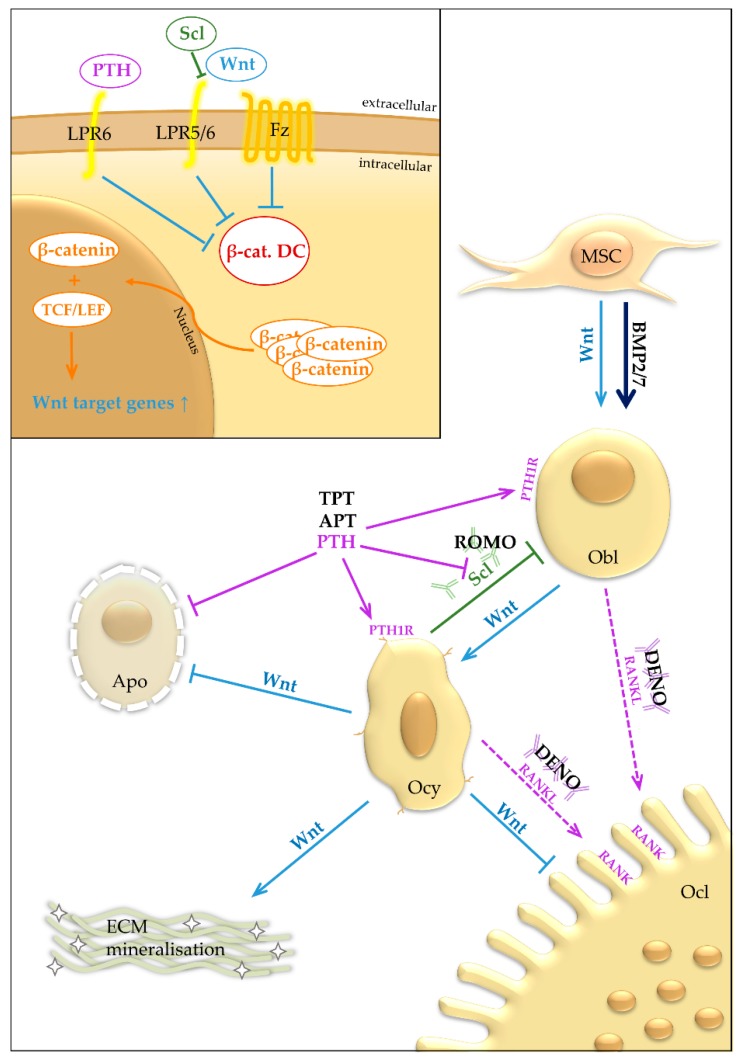
Top left: Wnt binds to the Frizzled receptor (Fz) and the LRP5/6 co-receptor. LRP5/6 and Fz deactivate the β-catenin destruction complex, which leads to accumulation of β-catenin. β-catenin translocates into the nucleus, where it regulates transcription of Wnt target genes with TCF/LEF. Sclerostin inhibits binding of Wnt to LRP5/6. PTH binds to LRP6 and causes an Wnt-independent deactivation of the β-catenin destruction complex. *bottom:* Wnt promotes the osteoblastic lineage and inhibits osteoclastogenesis and apoptosis. BMP is a strong promoter of osteoblastic differentiation. PTH acts through the PTH1R receptor in the osteoblastic lineage and has an inhibiting effect on sclerostin expression. APT and TPT work through selective activation of PTH1R activation. ROMO binds sclerostin. DENO binds RANKL and prevents RANK activation. Apo, Apoptosis; APT, Abaloparatide, parathyroid hormone-related protein analogue; β-cat. DC, β-catenin destruction complex, targets β-catenin for ubiquitination and subsequent degradation in the proteasome; BMP2/7, Bone morphogenetic protein 2 and 7; DENO, Denosumab, monoclonal antibody against RANKL; ECM, Extracellular matrix; Fz, Frizzled receptor, G-protein coupled receptor, target for Wnt; LRP5/6, Low-density lipoprotein receptor-related protein 5 or 6; LRP6, Low-density lipoprotein receptor-related protein 6; MSC, Mesenchymal stem cell; Obl, Osteoblast; Ocl, Osteoclast; Ocy, Osteocyte; PTH, Parathyroid hormone; TPT, Teriparatide, peptide Fragment of PTH; PTH1R, parathyroid hormone 1 receptor; RANK, Receptor Activator of NF-κB; RANKL, Receptor Activator of NF-κB Ligand; ROMO, Romosozumab, monoclonal antibody against sclerostin; Scl, Sclerostin; TCF/LEF, T cell factor/lymphoid enhancer factor; Wnt, Wingless-related integration site/Wnt signalling pathway.

First, osteoclasts, originating from the hematopoietic/monocyte-macrophage lineage, are the only cells within the organism capable of bone resorption. Under the influence of specific cytokines, including receptor activator of nuclear factor kappa-B ligand (RANKL) and macrophage colony-stimulating factor, osteoclast progenitors fuse to form multinucleated osteoclasts, which attach to the bone surface and commence resorption [1]. A combination of lysosomal enzymes and hydrogen ions is used to break down the organic and the mineral phase of the bone matrix, respectively, resulting in resorption pits called Howship Lacunae [2]. Second, bone-forming osteoblasts are derived from mesenchymal stem cells through the activation of specific transcription factors including activating transcription factor 4, osterix and runt-related transcription factor 2 (Runx2) [3]. The differentiating osteoblasts migrate to the site of bone resorption and fill the Howship Lacunae by first depositing primarily new collagen. This non-mineralized bone matrix later mineralizes to form woven bone which is subsequently remodelled to yield mature, biomechanically stable lamellar bone [4]. Thereafter, osteoblasts either undergo apoptosis, flatten and become a bone-lining cell or further differentiate into osteocytes. Osteoblast-osteoclast communication is enabled through cell-cell contact, cytokines and extracellular matrix interaction [5,6]. Osteoblasts are capable of modulating bone resorption, whereas osteoclasts can affect the formation of new bone. Finally, osteocytes represent the most abundant cell type in mature bone. These cell types are embedded within the bone matrix and are considered to play a role in bone remodelling by transmitting signals to other bone cells regarding mechanical stress. One of most important osteocyte-derived signals is the peptide sclerostin [7]. In the bone microenvironment sclerostin inhibits Wnt/β-catenin signals, which is known to promote bone formation and to suppress bone resorption. In this way, sclerostin is thought to inhibit bone apposition and to activate bone resorption. Mechanistically, sclerostin inhibits the binding of Wnt ligands to their respective receptor complexes and therefore leads to decreased intracellular β-catenin, the key effector mediator of the Wnt pathway (Figure 1).

The activity of bone cells is influenced directly or indirectly by a large variety of different factors. Local factors including cytokines, chemokines and growth factors among others, are expressed and secreted by cells within the bone microenvironment and exert auto- and/or paracrine effects governing bone turnover. A large array of different systemic factors including hormonal signals have been demonstrated to regulate bone metabolism, for example parathyroid hormone and oestrogen which play a crucial role in the balance between bone formation and bone resorption [1]. 

In a healthy organism, the processes of bone resorption and formation are tightly regulated, resulting in the maintenance of sufficient bone mass with adequate structure and mechanical quality. If this balance is disturbed, osteoporosis may develop, which represents the most prevalent bone disease worldwide [8]. In most cases, osteoporosis is caused by increased bone resorption with insufficient bone formation, resulting in an increased fracture risk with high socioeconomic costs. The term osteoporosis was first used in the 19th century to describe abnormally hollow bones in cadavers [9]. Osteoporosis, as it is defined by the World Health Organization today, is a decrease of bone mineral density (BMD) measured at the lumbar spine or hip of at least 2.5 standard deviations from the mean of a healthy reference population. Additionally, a clinical method of diagnosis has been proposed by the National Bone Health Alliance Group not solely relying on BMD measurement [10,11] but also including the recommended criteria of specific fracture occurrence and fracture risk score (i.e., FRAX, see below), providing an alternative basis for osteoporosis diagnosis.

Patients with osteoporosis have a disrupted bone architecture, a lower quality of bone tissue and, as a result, compromised bone strength and increased risk of fracture [8,12]. Osteoporosis affects an ever-increasing number of people in the aging population of modern society. According to the United States Centre for Disease Control, approximately 16.2% of adults over the age of 65 have osteoporosis and 48.3% of the same population exhibit a low bone mass (decrease of BMD between 1.5 and 2.5 standard deviations). Women over the age of 65 have a 5-times higher prevalence of osteoporosis than men, while only showing a much smaller increase in the prevalence of low bone mass. Aside from postmenopausal osteoporosis, caused by a decrease in oestrogen and senile osteoporosis, there are multiple causes for secondary osteoporosis. The most common cause of secondary osteoporosis is represented by glucocorticoid-induced osteoporosis (GIOP). Continuously increased glucocorticoid levels result in a decrease in osteoblast differentiation and function and an increase in osteoclastogenesis [13]. Importantly, the sole evaluation of BMD is not sufficient to assess fracture risk in GIOP, as it fails to reflect the disruption of bone architecture and increased risk of falls.

As stated above, a major complication of osteoporosis is an increase in fracture risk. Every fifth man and every other woman over the age of 50 will sustain a fracture due to increased bone fragility in their lifetime [8]. Fractures in elderly patients, depending on localization, morphology, comorbidities and healing potential, can lead to lasting disability and death. Fractures which are attributable to osteoporosis, are most commonly femoral neck fractures, vertebral fractures, distal radius fractures and pelvic fractures, followed by femur shaft fractures, humerus fractures and rib fractures [14]. Factors that increase fracture risk in osteoporotic patients include but are not limited to age, history of fall, previous fracture, diabetes, smoking, rheumatoid arthritis, long-term glucocorticoid use and alcohol use [8,15,16]. Scores have been developed to evaluate the fracture risk in osteoporotic patients, for example the most widely known Fracture Risk Assessment Score (FRAX), which takes a selection of nine risk factors into account [9,17]. Although widely used, the benefit of these scores is controversial and thus has not been established into general guidelines [18]. The mortality after osteoporotic fracture is dependent on the type of fracture, treatment and postoperative mobility, as well as BMI and comorbidities [19]. In the case of hip fractures, fewer than half of the hospitalized patients recover pre-fracture competence in their activities and mortality is as high as 36% within the first year following fracture [20]. Based on the high prevalence of osteoporosis in modern society, a 50-year-old woman’s lifetime risk of dying from a hip fracture was reported equal to her risk of dying from breast cancer [21].

In the light of these facts it is apparent that osteoporosis requires effective treatment. The foundation of treatment and prevention of osteoporosis has been reviewed elsewhere and includes weight-bearing exercises, fall avoidance and adequate nutrition to ensure sufficient calcium, vitamin D and protein intake [22]. These general measures, however, are not effective in all patients, especially in geriatric patients confined to nursing homes and in patients who have previously experienced an osteoporotic fracture and thus may require additional pharmacologic treatment regimes. The current pharmacological therapy aims at correcting the imbalance between bone resorption and formation at the level of osteoclasts and osteoblasts, thereby decreasing the risk of fracture events. A number of pharmacologic agents have been identified to lower fracture risk in both experimental and clinical studies. These pharmacological agents can be broadly subdivided into two principal groups: those decreasing bone resorption (by inhibiting osteoclast activity) and those increasing bone formation (by enhancing osteoblast activity).

### 2.1. Osteoporosis—Antiresorptive Therapy

It appears evident that the inhibition of bone resorption prevents loss of bone mass and architecture, explaining the fact that antiresorptive drugs represent a widely used substance class. Antiresorptive agents including bisphosphonates and the monoclonal antibody to RANKL (denosumab) target the generation, function and survival of osteoclasts and thus reduce the rate of bone resorption. As bone formation is coupled to bone resorption, inhibition of bone resorption is followed by a decrease in osteoblast activity. While this is initially associated with an increase in bone mineral density and some improvement of structural and material properties of bone tissue, increasing evidence points towards an association of long-term suppression of osteoclast activity with increased microdamage accumulation and an alteration in both bone mineralization and collagen formation [23]. Although antiresorptive drugs in general display a low rate of adverse effects, the suppression of bone turnover may explain necrosis of the jaw and the occurrence of atypical fractures of the femur which can be observed in patients with high-dose or long-term bisphosphonate usage, respectively [24,25]. Therefore, as antiresorptive agents fail to adequately restore bone mass and bone quality, there is a continued interest in the identification of molecular targets which stimulate osteoblast activity and result in an increased bone mass with restored skeletal architecture.

### 2.2. Osteoporosis—Anabolic Therapy

In principle, stimulating bone formation by pharmacologic means (anabolic therapy) can increase bone mass to a greater extent than antiresorptive drugs. While there is a variety of different antiresorptive agents employed in every day clinical practice (e.g., oestrogen, selective oestrogen receptor modulators, bisphopshonates, denosumab), the only currently available treatment regimen to stimulate bone formation is represented by daily injections of parathyroid hormone (PTH) or one of its analogues such as teriparatide and abaloparatide.

#### 2.2.1. PTH—Teriparatide and Abaloparatide

In a healthy organism, PTH functions as an essential endocrine regulator of calcium and phosphate concentrations in the extracellular space, which is crucial for maintaining serum and urinary calcium levels within the physiological range. Chronically elevated PTH levels, as observed in primary and secondary hyperparathyroidism, cause a high bone-turnover state with bone resorption exceeding bone formation, ultimately resulting in osteoporosis [26]. However, daily injections of PTH (intermittent PTH or iPTH) or its peptide fragment PTH1–34 (teriparatide) with recurrent, temporary rises in serum concentration, primarily stimulate bone formation and only to a minor extent bone resorption [27]. This results in a net effect of increased bone mass, improved bone microarchitecture and increased mechanical strength. 

In skeletal tissue, PTH primarily binds to and exerts its biologic effects through the parathyroid hormone 1 receptor (PTH1R). Among other cell types, this G protein-coupled receptor is expressed in mesenchymal stem cells, osteoblasts and osteocytes but not in osteoclasts (Figure 1). It is now understood that the catabolic (i.e., pro-resorptive) effect of PTH is primarily mediated through an increased expression of RANKL and the decreased production of its decoy receptor osteoprotegerin (OPG) in osteoblasts and their precursors and possibly also in osteocytes [26]. Although the precise molecular mechanism by which PTH stimulates bone formation is not entirely clear to date, previous studies demonstrated that PTH increased the proliferation and differentiation of osteoblasts and their precursors both in vitro and in vivo. Moreover, PTH was shown to inhibit osteoblast apoptosis and to activate bone lining cells. Mechanistically, transactivation of Runx2, the transcription factor crucial for osteoblast differentiation, is activated by PTH through cAMP/protein kinase A [28]. Moreover, ERK1/2-mitogen-activated protein kinase and phosphatidylinositol phosphate signalling pathways are also activated by PTH, which results in an enhanced osteoblast proliferation [29]. 

Another significant effect of PTH is the activation of the Wnt signalling pathway in cells of the osteoblast lineage, including osteoblasts and their precursors, as well as osteocytes [30]. Wnt ligands bind to receptors of the Frizzled family together with co-receptors of the low-density lipoprotein receptor-related protein (LRP) family, LRP5 and LRP6 [31]. This results in the activation of canonical signalling cascades and the stabilization of cytosolic β-catenin, a key effector mediator of the Wnt pathway. After translation into the nucleus, β-catenin forms a complex with the T cell factor/lymphoid enhancer factor (TCF/LEF) family of transcription factors and proceeds to interact with the genomic DNA to regulate the transcription of Wnt target genes [31]. PTH was shown to increase β-catenin levels in cells of the osteoblast lineage and thus stimulate osteoblast proliferation and differentiation [32]. Another study found that PTH, once bound to PTH1R, is also capable of directly complexing with LRP6, resulting in Wnt ligand-independent activation of β-catenin activation [23,33].

PTH may not only stimulate bone formation through a direct effect on Wnt signalling in osteoblasts but also indirectly through reducing sclerostin production by osteocytes [7,34]. Sclerostin represents an osteocyte-specific protein, which potently antagonizes Wnt signalling in bone cells [35]. This hypothesis results from the observations that PTH suppresses the expression of sclerostin in bone tissue, that PTH levels inversely correlate with sclerostin levels in healthy women and that women treated with iPTH display decreased serum concentrations of sclerostin [36,37]. Initial experimental studies revealed no increase in bone mass in the distal femur of both sclerostin-deficient and sclerostin-overexpressing mice receiving iPTH [38]. However, other studies showed that iPTH increases both bone formation and resorption in both wildtype and sclerostin-deficient mice [39]. Furthermore, iPTH significantly increased the trabecular thickness and mineral apposition rate in sclerostin-deficient mice, indicating that iPTH stimulates bone formation independently of sclerostin suppression [39]. This uncertainty regarding the role of sclerostin in the osteoanabolic effect of iPTH lies within the altered baseline bone density, which is characteristic of mice either lacking or overexpressing sclerostin and further studies are warranted to dissect the exact molecular mechanism responsible for the therapeutic effect of iPTH.

Although iPTH or teriparatide primarily stimulate bone formation through its high affinity for the R0 conformation of the PTH1R, a gradual increase in bone resorption can be observed during prolonged usage [40]. Therefore, the clinical use of iPTH and teriparatide action is based on its effect of stimulating bone formation before it enhances bone resorption, the period when they are maximally anabolic (anabolic window). In the case of PTH, the anabolic window lasts approximately 18 to 24 months, before bone resorption exceeds bone formation and no net increase in bone mass can be achieved, limiting its therapeutic use to a maximum of 2 years [41].

In order to possibly prolong the anabolic window, abaloparatide, a structurally related agent has been developed and recently approved by the FDA for the treatment of postmenopausal osteoporosis. Abaloparatide is a synthetic analogue of parathyroid hormone-related protein (PTHrP) which binds transiently to the RG conformation of PTH1R and also requires daily subcutaneous injections. Experimental studies demonstrated that abaloparatide increases trabecular thickness and improves trabecular microstructure [42]. In a phase 3 clinical trial with 2463 ambulatory postmenopausal women, of which 1901 completed the study, abaloparatide was shown to reduce vertebral and non-vertebral fractures compared to placebo or teriparatide [43]. According to currently available data, abaloparatide reduces the number needed to treat for prevention of non-vertebral, clinical and major osteoporotic fractures compared to teriparatide [44]. Nonetheless, the claim that the anabolic effect is accompanied by less bone resorption with abaloparatide than teriparatide, thus widening the anabolic window, still requires further evidence [45]. Abaloparatide was approved by the FDA in April 2017. However, a higher risk of select adverse effects including cardiovascular events when compared to teriparatide have resulted in the refusal of the marketing authorization by the European Medicines Agencies so far [46].

#### 2.2.2. Sclerostin-Neutralizing Antibody—Romosozumab

Searching for novel targets to increase bone formation, researchers soon became interested in a rare, autosomal-recessive form of a high bone mass disorder, which resulted in the identification of sclerostin as a key regulator of osteoblast activity. Patients with sclerosteosis—a loss of function mutation—or Van Buchem disease—a genetic mutation affecting sclerostin expression—display high bone mass with excellent biomechanical stability due to an excessive osteoblast activity [47]. Similarly, mice lacking functional sclerostin protein display a striking high bone mass phenotype, whereas transgenic mice over-expressing sclerostin are osteoporotic [48]. Further mechanistic studies demonstrated that sclerostin, secreted primarily from osteocytes within the bone microenvironment, reaches the bone surface through osteocyte canaliculi, where it inhibits co-receptor localization with Frizzled receptors through binding LRP5 and/or LRP6 [7,35,49]. Activation of Wnt signalling is thus inhibited, resulting in decreased osteoblastogenesis and bone formation. In addition, sclerostin was demonstrated to promote bone resorption by increasing the production of RANKL in osteocytes [50]. Although the exact mechanism of action is still not fully clarified to date, it is undoubted that sclerostin is primarily produced by osteocytes and that it acts as an anti-osteoanabolic molecule (Figure 1).

As a rational consequence of these observations, the therapeutic effect of inhibiting sclerostin with neutralizing antibodies in various animal models was subsequently tested. Data from these experimental studies showed a consistent effect of sclerostin immunoneutralization to increase bone formation, bone mass and biomechanical stability at various skeletal sites [51,52]. These results led to the development of romosozumab, a highly specific, monoclonal antibody against human sclerostin which is applied subcutaneously once every month.

Phase III clinical trials (FRAME and STRUCTURE) in female patients suffering from postmenopausal osteoporosis have shown that romosozumab increases bone mineral density at the lumbar spine and hip and reduces the risk of vertebral and clinical fractures in comparison with placebo [53,54]. Romosozumab reduced the risk of vertebral, non-vertebral and clinical fractures in comparison with the bisphosphonate alendronate in women with severe osteoporosis (ARCH) [55]. This was accompanied by an increase in the markers of bone formation, whereas the markers of bone resorption decreased, indicating dual action (i.e., stimulation of bone formation and inhibition of bone resorption) of romosozumab. At present, the approval of romosozumab by the authorities is awaiting further investigations of a potential increased risk of serious adverse effects including cardiovascular events, which has been associated with romosozumab treatment in the ARCH study [55].

#### 2.2.3. Future Perspectives

Apart from the agents discussed above, various cytokines, chemokines, growth-factors and other signalling molecules have been identified to be of crucial importance in regulating bone formation [56,57,58] and may thus represent suitable targets to augment osteoblast function. Their use as bone-anabolic agents, however, is often hindered by the fact that tissue-specific delivery at sufficient dosage cannot be achieved [58]. As an alternative, gene therapy or transfer offers an attractive technology, which could potentially overcome these limitations. Although not tested in humans, several experimental studies with animal models have proven the potential efficacy of this novel approach. Exogenous genetic material is introduced in order to modify or correct cell differentiation or function. Targeted delivery and transcription of genes encoding critical regulators in bone remodelling including BMPs, PTH or OPG has proven efficient to treat experimental osteoporosis [59,60,61,62,63,64,65,66,67]. The protective effect was not limited to the bones which were intramedullary injected with the respective vectors but also in other bones of the same animal. Moreover, based on the growing understanding of the role of microRNA (miRNA) in the epigenetic regulation of osteoporosis and bone metabolism [68], targeted activation or inactivation of bone-specific miRNA could represent yet another molecular therapy to boost osteoanabolic responses in the skeleton. Although further work is required to fully comprehend the potential clinical implications and to exclude potential serious adverse effects, this encourages the further development of gene therapy as a novel approach to stimulate bone formation in osteoporosis.

## 3. Fracture Healing—Impaired Bone Regeneration

Bone tissue is not only continually remodelled by the combined and tightly regulated activity of bone cells but also has the remarkable capacity for scar-free repair following fracture. The processes governing bone turnover in health and disease are also effective during bone regeneration, as fracture healing can be regarded to represent a juxtaposition of tissue formation (anabolism) and tissue resorption (catabolism or remodelling). These concepts are useful for understanding bone repair and have led to the evaluation of osteoporosis drugs for the treatment of impaired fracture healing. 

Fracture healing or bone regeneration, results from a complex interplay of cellular and molecular signalling events that reiterate embryonic skeletal development. Traditionally, fracture healing is subdivided into four main phases that show a significant degree of overlap: (1) inflammatory phase, (2) soft callus phase, (3) hard callus phase and (4) remodelling phase [69]. Bone regeneration starts with an inflammatory response and hematoma formation caused by the disruption and leakage of the bone marrow and damage to the vascular and soft tissue. A hypoxic sub-phase promotes revascularization. This is followed by the formation of a soft fibro-cartilaginous matrix, consisting primarily of fibroblasts and chondrocytes, which provides a certain degree of mechanical stability at the fracture site and acts as a template for the hard callus. Due to the combined activity of osteoclasts and osteoblasts, the soft callus is gradually replaced by hard callus during the osteogenic phase, resulting in irregular woven bone with high vascularization. Finally, the woven callus is replaced by lamellar bone which resembles the original cortical and trabecular form of mature bone.

Fracture healing is an evolutionary highly conserved process which functions effectively and efficiently without significant complications in the majority of affected patients. However, in up to 10–20% of patients with fractures, impaired bone regeneration including fracture non-union can be observed, despite the considerable progress in the advance and optimization of surgical fracture care [70]. Non-union is defined as a fractured bone, for which a minimum of nine months has elapsed since the injury and for which there have been no signs of healing for three months. Aside from the high medical costs associated with the treatment of non-unions, patients suffering from delayed- or non-union are frequently unable to follow their occupation during the treatment process [71]. A large range of different factors has been identified to be associated with impaired bone regeneration, including intrinsic factors, such as the age and gender of the patient and extrinsic factors, such as the location and extent of displacement of the fracture. Non-union presents an ongoing therapeutic challenge and, similar to osteoporosis, is often associated with significant morbidity, resulting in decreased quality of life in affected patients and high socioeconomic costs. 

### 3.1. Impaired Fracture Healing—Antiresorptive Therapy

The use of bisphosphonates in osteoporosis for the prevention of fragility fractures is well established, their value in promoting fracture healing and in preventing and treating non-union much less so [72,73,74]. In animal studies, bisphosphonates were shown to cause an increase in callus volume and bone mineral content during primary enchondral ossification, while causing delayed remodelling of the fracture callus [75,76]. They increase the bone-implant contact after surgical fixation of the fracture, however they do not appear to affect the healing rate or time [77,78]. In clinical studies bisphosphonates have been shown to increase overall BMD and time to union after distal radius fracture [71,79]. Similar to animal studies however, bisphosphonates do not reduce time to consolidation of the fracture or the rate of healing and bolus bisphosphonate therapy 2 weeks after surgery has been demonstrated to increase BMD in the hip and to significantly reduce overall mortality [80].

The monoclonal antibody denosumab binds to RANKL, prevents it from binding to its receptor RANK on the cell surface and therefore inhibits osteoclast recruitment and differentiation. Similar to bisphosphonates, denusomab has been shown to increase callus formation and delay remodelling in animal studies, however the formed callus seems to have better biomechanical properties compared to bisphosphonate treatment [79]. In the clinical trials conducted to date, denusomab did not delay fracture healing in patients primarily receiving antiresorptive therapy for osteoporosis [81]. However, clinical studies on the effect of denusomab on impaired fracture healing including delayed or non-union are insufficient to allow for clinically relevant conclusions and warrant further studies.

### 3.2. Impaired Fracture Healing—Anabolic Therapy

As osteoclast function is required to remove necrotic bone fragments and the cartilaginous tissue intermediate during bone regeneration, it is assumed that the stimulation of bone formation is more favourable to improve bone regeneration than the inhibition of bone resorption. Based on the anabolic effect of iPTH, teriparatide and abaloparatide in intact bone, this has led researchers to investigate their use for the prevention and treatment of impaired fracture healing. 

#### 3.2.1. PTH

Both iPTH and teriparatide have been shown to promote fracture healing in animal studies employing various species [26]. Callus developing under iPTH treatment has been shown to mature faster and to exhibit superior biomechanical properties compared to controls [82]. iPTH promoted accelerated bone formation in a murine open fracture model, although there was no increase in the rate of bone union [83]. Teriparatide has also been shown to increase chondrocyte differentiation and recruitment and therefore to enhance enchondral ossification [30]. Furthermore, iPTH caused a 2 to 3-fold increase in regulatory T-cell populations in mice, which in turn were previously shown to promote callus formation by balancing the excessive inflammatory reaction observed during the early stages of fracture repair [84,85]. A recent study comparing the effects of teriparatide and abaloparatide on bone healing in rats found both drugs to improve fracture healing but in the employed models the potency per µg of abaloparatide seemed lower than the relation reported from the human osteoporosis trial (ACTIVE) [45,86]. 

Because most animal studies used PTH or its analogues in supraphysiological doses, associated with the potential risk of osteosarcoma development following long-term application, there were significant concerns that clinical studies using only physiological doses would not show the desired results for treatment efficacy [87]. Available clinical studies employing varying protocols of dosing, timing and duration of application for fracture treatment have provided conflicting results [88]. In this regard, it is worth mentioning that PTH may not only be applied systemically but also locally. Animal studies investigating the local delivery of PTH or teriparatide via various scaffolds implanted into bone defects have shown promising results and reported a superior rate and degree of ossification [89]. However, similar to the systemic route of application, insufficient understanding regarding optimal dosing and timing has prevented the use of locally applied PTH or its derivatives in clinical practice so far. 

#### 3.2.2. Bone Morphogenetic Proteins

One family of peptides, on which more profound information regarding pharmacologic application and clinical value to boost bone regeneration is available, is represented by bone morphogenetic proteins (BMP). BMPs are a family of cytokines pertaining to the TGF-β superfamily and function as key regulators of tissue development in embryonic and adult animals. BMPs were first discovered in 1965 for their capacity to induce ectopic bone formation [90]. To date, over 30 different BMPs have been described and associated with pleiotrophic functions in regulating a wide range of different cell types, including mesenchymal stem cells and cells of the osteoblast and chondroblast lineage required for bone regeneration. The concentration of BMPs and their function varies greatly throughout the process of fracture healing. BMP-2, -4 and -7 were found to be expressed at high levels during the early stages of fracture repair around the periosteum and to potentiate the differentiation of mesenchymal stem cells into chondroblasts and osteoblasts [91,92]. In contrast, BMP-3 is one of the few BMPs expressed in osteoclasts and can be considered to function as an antagonist of most osteogenic BMPs [92]. Local delivery of BMPs has shown promising results in animal studies for spinal fusion and fracture healing. Recombinant human BMP-2 (rhBMP-2) was subsequently approved by the FDA in the early 2000s for open tibial fractures, anterior interbody fusion in the lumbar spine and subsequently maxillary sinus and alveolar ridge augmentation after tooth extraction to fill resulting defects; rhBMP-7 was approved for open tibia fractures. Multiple series of off-label use randomized clinical trials were published, including cervical spinal fusions, radius fractures and non-union [92]. 

In bone defects, BMPs promoted healing when used in combination with a variety of scaffolds and autologous or allogenic grafts. This was shown in both small and large animal models with cranial and maxillary defects, as well as with segmental bone defects otherwise resulting in non-unions [93,94,95]. However, clinical testing of locally applied rhBMP has revealed potential detrimental side effects, such as heterotopic ossification, inflammation and oedema, in addition to osteolysis when used in high concentrations. Severe clinical complications like swelling in cervical spinal fusion causing airway obstruction and segmental spinal collapse due to increased bone resorption, have caused a re-evaluation of the use of BMPs for enhancing bone fracture healing [96,97,98]. Glaeser et al. have however recently managed to reduce inflammation and swelling while causing a stimulation in BMP-2 mediated bone formation through application of the NEMO binding domain peptide (NBD) with BMP-2, opening the possibility for reduction of the complications associated with clinical use of BMP-2 [99]. NBD inhibits the activation of NF-κB, a central regulator to the inflammatory response. The combination of adjuncts with lesser doses of BMPs may provide a future perspective for clinical applications. It is noteworthy that none of the hitherto tested BMPs is approved for systemic application or osteoporosis therapy, based on their short half-life and the aforementioned adverse effect. 

#### 3.2.3. Sclerostin-Neutralizing Antibodies

In conjunction with rhBMP-2 anti-sclerostin antibodies were reported to improve bone regeneration in a rat femoral defect model when compared to rhBMP-2 alone [100]. However, in a study on segmental defects in rats without additional BMP, the application of anti-sclerostin antibody did not enable bony bridging and solely induced an osteoanabolic response in the surrounding intact bone, which is explained by its lack of osteoinductive potential [101]. A recent study demonstrated that Sost-deficient mice, which do not express sclerostin protein, are capable of bridging critical-size calvarial bone defects, which otherwise fail to heal in wild-type mice [102]. Based on the currently available data, the anti-sclerostin antibody romosozumab developed for the treatment of osteoporosis, may have possible applications in the treatment of skeletal defects in bones with intramembranous ossification such as the skull. Similar to PTH and its related analogues, further clinical studies employing different pharmacologic timing and dosing are required in order to evaluate the clinical value of inhibiting sclerostin during fracture repair.

#### 3.2.4. Future Perspectives

Due to the potential side effects associated with the systemic application of a number of substances with high potential for bone regeneration, some research groups have focused on establishing local delivery methods to defect sites. Both non-genetic methods, such as conjugating oligoaspartic acid, which has a high affinity for hydroxyapatite in fracture sites and promotes elevated concentrations of the chosen agent within the fracture site, and methods using gene therapy for enhancing local transcription of growth factors have been described [103]. For example, a number of research groups have tried to find alternative methods to modulate the BMP-2 signalling pathway within the fracture site using viral vectors or copolymer-protected gene vectors [104,105]. However, these approaches are purely experimental at this stage and warrant further investigation for their clinical use to promote bone regeneration.

## 4. Conclusions

Based on the current demographic development, the number of patients with diseases of the musculoskeletal system including osteoporosis and impaired fracture healing is expected to rise steadily. The understanding of the complex cellular and molecular interactions that govern bone metabolism and bone regeneration in health and disease has given rise to novel compounds with high therapeutic potency and a potential low risk for adverse effects. The nature of osteoporosis and impaired bone regeneration, as well as the presence of different comorbidities in affected patients, may require individualized treatment regimens employing more than just one bone drug to achieve the best possible outcomes. The further development and study of therapeutic approaches targeting bone formation, rather than bone resorption, fulfills an essential clinical need, as treatment options for reversing bone loss and promoting bone regeneration are currently limited. 

## Abbreviations

ACTIVE	Abaloparatide Comparator Trial In Vertebral Endpoints Trial
ARCH	Active-Controlled Fracture Study in Postmenopausal Women with Osteoporosis at High Risk
BMD	Bone mineral density
BMI	Body mass index
BMP	Bone morphogenetic protein
cAMP	cyclic Adenosine monophosphate
ERK1/2	Extracellular-signal Regulated Kinase 1 and 2
FDA	United States Food and Drug Administration
FRAME	Fracture Study in Postmenopausal Women with Osteoporosis
FRAX	Fracture Risk Assessment Score
GIOP	Glucocorticoid-induced osteoporosis
iPTH	Intermittent PTH
LRP5/6	Low-density lipoprotein receptor-related protein 5 or 6
LRP6	Low-density lipoprotein receptor-related protein 6
miRNA	Micro RNA
NBD	NEMO binding domain peptide
NEMO	NF-kappa-B essential modulator
NF-κB	Nuclear factor “kappa-light-chain-enhancer” of activated B-cells
OPG	osteoprotegerin
PTH	Parathyroid hormone
PTH1-34	Teriparatide, peptide Fragment of PTH
PTH1R	parathyroid hormone 1 receptor
PTHrP	parathyroid hormone-related protein
RANK	Receptor Activator of NF-κB
RANKL	Receptor Activator of NF-κB Ligand
rhBMP	Recombinant BMP
Runx2	Runt-related transcription factor 2
Scl	Sclerostin
STRUCTURE	An Open-label Study to Evaluate the Effect of Treatment With Romosozumab or Teriparatide in Postmenopausal Women
TCF/LEF	T cell factor/lymphoid enhancer factor
Wnt	Wingless-related integration site/Wnt signalling pathway
WT	Wild type
